# Insights into cerumen and application in diagnostics: past, present and future prospective

**DOI:** 10.11613/BM.2017.030503

**Published:** 2017-10-15

**Authors:** Engy Shokry, Nelson Roberto Antoniosi Filho

**Affiliations:** Laboratório de Métodos de Extração e Separação, Instituto de Química, Universidade Federal de Goiás, Goiânia, Brazil

**Keywords:** cerumen, laboratory diagnosis, metabolomics, proteomics, genomics

## Abstract

Cerumen or earwax is an emerging bio-fluid in clinical diagnosis that has been very little exploited during the past decades in spite of its high diagnostic potential. It is highly abundant in diagnostic biomarkers such as genetic material, lipids, proteins, chemical elements, internal and external metabolites (*e.g.* hormones, volatile organic compounds, amino acids, xenobiotics *etc.*) reaching earwax from the blood circulation. Thus, it is able to reflect not only physiology, pathophysiology of the human body but can also detect recent and long term exposure to environmental pollutants, without the need of invasive blood tests and in the same time overcoming many disadvantages faced by using other diagnostic biological fluids. This review discusses the biology, functions, chemistry of earwax, past and current approaches for the study of its chemical composition, emphasizing how a detected variation in its composition can offer information of high clinical value, which can be useful in diagnosis of many diseases such as metabolic disorders and tumours as well as in forensic applications. It also presents details about techniques of sample collection, storage, and analysis. Moreover, it highlights concerns about the use of earwax for diagnostic purposes, which should be addressed to make earwax diagnostics a reality in the future.

## Introduction

Cerumen, commonly referred to as earwax, is a rich biological fluid that has distinct advantages as a biomonitoring medium of a high diagnostic potential. Although many studies have been concerned with the elucidation of the chemical composition of cerumen, the literature shows that until today very little attention has been dedicated to the analysis of cerumen with the aim of laboratory diagnosis. Cerumen, being composed of a large diversity of biomarker compound classes including lipids, proteins, amino acids (AA), carbohydrates, volatile organic compounds (VOC), chemical elements in addition to hormones, antibodies, enzymes and their products, makes it a reflection of the physiological functions of the body and a potential alternative biological matrix. This review provides an overview of the biology, functions of cerumen, past and current investigations performed on its chemical composition and its applications in laboratory diagnosis with emphasis on pre-analytical, analytical, and post analytical aspects. It also highlights its advantages and limitations in comparison to classical fluids.

## Composition and functions of cerumen

Cerumen is a waxy substance secreted by ceruminous “apocrine sweat” glands located subcutaneously in the external ear canal (*1*, *2*). Ceruminous glands in combination with the sebaceous glands produce earwax which is therefore, considered a mixture of sweat secretions and fatty material from the sebaceous glands (*1*, *2*). Regarding the chemical composition, it is composed of fatty acids, alcohols, ceramides, wax esters, triacylglycerols, long chain hydrocarbons, and cholesterol precursors as lanosterol, squalene, and cholesterol which are the final products in the hyroxymethylglutaryl-CoA (HMG-CoA) reductase pathway (*3*) with physical consistency ranging from wet, sticky and yellow or brown to dry, crumbly and white or greyish. Earwax production is affected by some factors such as working conditions, climate, and even increased cholesterol concentrations can block the HMG-CoA reductase pathway by negative feedback (*2*, *4*, *5*).

The earwax phenotype is determined by two alleles at a single gene termed as *ABCC11* gene (*6*). A single-nucleotide polymorphism (SNP) in this gene encodes an ATP-driven efflux pump protein responsible for the variation in the apocrine gland secretion which affects the earwax type being wet or dry as well as the axillary odour commonly called “underarm odour” (*6*, *7*).

The earwax phenotype was also linked to ethnicity/race, where the dry type is commonly prevalent in East Asians (95%) but rare in Europeans and Africans (3%). A mixed rate of dry and wet types with dry wax incidence of 30-50% is seen in populations of Native North Americans, the Pacific Islands, Central Asia, Turkey and those of Asian ancestry (*8*, *9*).

Among its main functions is to moisten, clean, lubricate, and protect the skin of the human ear canal, in addition to acting as an antibacterial maintaining the environment in the ear canal acidic and a barrier against foreign substances as water, insects and dust (*10*). Moreover, it can provide important information about an individual including race, ethnicity, gender, diseases, food eaten and exposure to surrounding environmental pollutants (*11*).

## Cerumen and implications to health, diagnostics and forensics

### Cerumen sampling, transport and preservation

Cerumen, being secreted inside the ear canal, is protected against external contamination which is a serious problem limiting the diagnostic potential of many biological samples. Despite this fact, certain precautions must be considered during sample collection to maximize the credibility of data obtained upon its analysis as: performing hand hygiene, using disposable gloves and apron during sample collection, swabbing/removing cerumen from the inner portion of the ear as opposed from the lobes (where soap/shampoo flakes are more prevalent), transferring the sample to vials/containers that are air tight and at low risk of cross contamination, putting a label with patient information; transportation to the laboratory for analysis (*12*, *13*).

**Figure 1:**
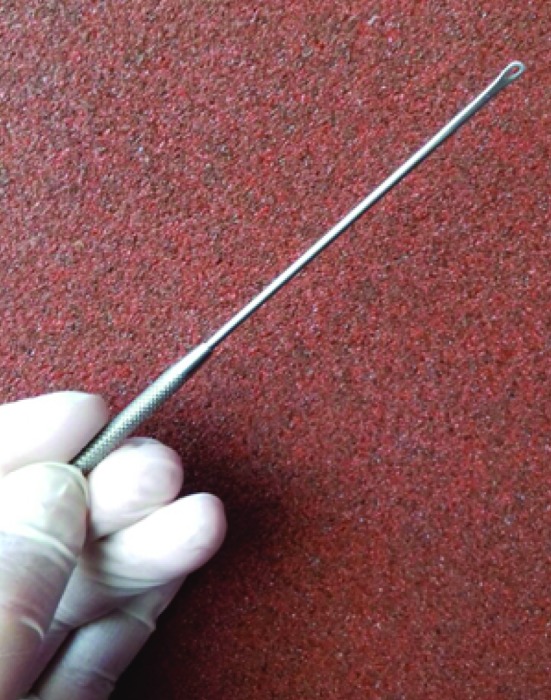
The Jobson-Horne probe, an effective curette for the removal of the dry and wet type earwax

For cerumen sampling, different techniques were applied depending on the quantity required for analysis including for instance ear swabs commonly applied in forensic testing, DNA paternity testing, *etc.* (*14*). Ear swabs could be performed at home by oneself placing a sterile swab (cotton buds or Q-tips) into the inner portion of the ear and gently rotating to collect the sample 2-3 times if possible (the more the better), whereas a full swab tip may be enough (*14*). Other techniques for sample collection were applied using tools such as sterilized metal scoops, plastic curettes, wooden spatulas, and the Jobson-Horne probe (*15*-*18*). Ear scoops (picks) are more efficient in the removal of the dry type of earwax, while the Jobson-Horne probe (Figure 1) being fenestrated is effective in the removal of either the dry or moist-type (*18*, *19*). Generally, use of ear picks is better done by a health professional to avoid risks of damaging the ear and causing infections (*20*).

The sampling time required to obtain samples with detectable concentrations involving casual cerumen is variable. Normally, the subjects involved are instructed not to clean the ears one week to ten days to allow for sample build up before collection and to bathe/shower with fragrance-free liquid soap/shampoo, and avoid any kind of perfume or fragrance, in order not to affect the earwax volatile composition (*21*). In case of fresh cerumen sample, first casual cerumen is removed from the ears of the study participants; then the ears are irrigated with water and wiped with cotton swabs moistened with an alcohol-ether (3:1) mixture then the fresh cerumen is collected 24 or 48 hours later (*21*).

Samples are stored in sterile airtight containers (vials, bottles, Eppendorf tubes, *etc.*) kept away from sunlight, chemicals or conditions that may catalyse sample degradation (*13*, *21*, *22*). As for sample transport and preservation, the conditions are dependent on the nature of the compounds being analysed. For instance, in case of ear swabs for DNA testing, the sample can be shipped to the laboratory in a paper envelope and stored somewhere safe at room temperature for up to six months (*14*). In case of analysis of volatile organic composition; samples are usually stored in a deep freezer at - 30 °C and analysed within one week to minimize the loss of the volatiles, while those used for lipidomics and proteomics are usually preserved at - 18 °C and - 80 °C, respectively (*15*, *17*, *21*). For samples that need immediate refrigeration/freezing, they can be kept in portable freezers with controllable temperatures available at the collection sites until transported to the laboratory (*16*).

Proposals were sought to enhance the use of dried biological samples, one of which is dried cerumen for clinical analyses with the aim of enhanced recovery of analytes and automated processing of dried specimen samples - particularly if elements of integrated sample preparation are necessary to preserve the recovery of integrity of a given biomarker class for later detection (*23*).

### Analysis of cerumen

Cerumen has been subjected to different kinds of analyses of compound classes such as lipidomic, proteomic, genomic and metabolomic analyses in addition to analysis of chemical elements, and xenobiotics *e.g*. drugs, foreign pollutants, *etc.* Details on selected applications of cerumen analysis in laboratory diagnostics including compound classes involved, populations tested, pre-analytical, analytical and post analytical aspects are shown in Table 1.

**Table 1:** Overview of the selected applications of cerumen analysis in laboratory diagnostics

**Approach**	**Population** **tested**	**Sample** **collectionn**	**Sample** **pretreatment**	**Method of analysis**	**Disorder**	**Biomarkers detected**	**Detected ranges**	**Conventional bio-fluids**	**Reference**
**Proteomics**	2 female, 3 male	Small metal scoop	1D-PAGE pre-fractionation, online SCX-fractionation	LC-MS/MS	chronic otitis externa, otomycosis, benign or malignant ear tumors, systemic diseases as (diabetic nephropathy, breast cancer)	zinc-alpha-2-glycoprotein	Qualitative	Plasma, saliva, urine	Feig *et al.* (2011)
**Genomics**	40 patients (18 females, 23 males, 17-57 years)	Sterile swabs	Homogenization in saline, then DNA extraction	Real-time PCR	Diagnosis of Hepatitis B infection	Hepatitis B virus DNA	4.2 x 10^2^ - 4.7 x 10^6^ copies per sample	Serum	Kalcioglu *et al* (2004)
	50 patients (21 females, 29 males, 20-40 years)	Sterile spoon /sterile swabs	Homogenization in saline, then DNA extraction	Real-time PCR	Diagnosis of Hepatitis B infection	Hepatitis B virus DNA	ND	Serum, saliva	Parizad *et al.* (2016)
	35 patients (21 females, 29 males, 21-70 years)	Sterile swabs	Homogenization in saline, then DNA extraction	Real-time PCR	Transmission of Hepatitis C infection	Hepatitis C virus RNA	no copies per sample	Serum	Bayindir *et al.* (2005)
**Metabolomics**									
**Volatile organic compounds**	8 patients (24-48 years)	ND	Desiccation by electric plate at 110^o^C	Change in weight	Allergic rhinitis	Total volatile compounds	28.4 ± 8.5 (43.8 ± 12.2 µg/g)*	Blood	Paiva *et al.* (1973)
	17 patients (7-61 years)	ND	Desiccation by electric plate at 110^o^C	Change in weight	Otoschlerosis	Total volatile compounds	31.1±10.4 (43.8±12.2 µg/g)*	-	Paiva *et al.* (1973)
	12 patients (46-72 years)	ND	Desiccation by electric plate at 110 ^o^C	Change in weight	Cancer	Total volatile compounds	30.7 ± 10.3 (43.8 ± 12.2 µg/g)*	Blood, urine, saliva, sweat	Paiva *et al.* (1973)
	Neonates (< 5 days)	Metal scoop	Solvent extraction and derivatization	GC, GC-MS	Maple syrup urine syndrome	Sotolone (4,5-dimethyl-3-hydroxy-2[5H]-furanone), a metabolite of isoleucine or allo-isoleucine	Qualitative (burnt sugar odour)	Urine	Kataoka *et al.* (2013), Liebich *et al.* (1983)
	2 females patients (61 years)	ND	Solvent extraction	Paper chromatography, visualization with 5% ammonium silver nitrate	Alkaptonuria	Homogentisic acid	Qualitative	Urine	Frohlich *et al.* (1973)
	26 patients (13 males, 13 females)	Plastic curette	No previous extraction	HS/GC-MS	Diabetes mellitus (Types 1 and 2)	Acetone, methoxyacetone, ethanol, isobutyraldehyde, hydroxyurea, acetic acid	Chemometric treatment of quantitative data	Blood, plasma, urine	Shokry *et al.* (2017)
**Approach**	**Population** **tested**	**Sample** **collectionn**	**Sample** **pretreatment**	**Method of analysis**	**Disorder**	**Biomarkers detected**	**Detected ranges**	**Conventional bio-fluids**	**Reference**
**Xenobiotics**	ND	ND	Solvent extraction	GC, GC-MS	Exposure to toxic chemicals	Lindane, chlordane, DDT, DDE, dieldrin, HCB, HCH (long term) exposure)	Detected (0.0 µg/g)*	Breath, blood (short term exposure), adipose tissue, breast milk (long-term exposure)	Lauwerys *et al.* (1991), Wang *et al.* (1988)
	3800 (35-54 years)	ND	Solvent extraction	GC, GC-MS	DDT, HCH induced cancer mortality	DDT, HCH	Detected (0.0 µg/g)*	Breath, blood, adipose tissue	Wang *et al.* (1988)
	10 males and 7 females (≥ 18 years)	Plastic curette	Direct extraction with methanol	UPLC- MS/MS	Administration of drugs of abuse or drug facilitated crimes	lacosamide	13.2 ng/mg (0.0 ng/mg)*	Blood, plasma, urine, saliva (short term), hair (long term)	Shokry *et al.* (2017)
						lamotrigine	9.5 - 115.0 ng/mg (0.0 ng/mg)*		
						carbamazepine	13.2 - 259.5 ng/mg (0.0 ng/mg)*		
						phenytoin	8.7 - 243.3 ng/mg (0.0 ng/mg)*		
						levitracetam	52.0 ng/mg (0.0 ng/mg)*		
						oxcarbazepine	5.0 - 326.5 ng/mg (0.0 ng/mg)*		
						valproic acid	186.5 - 4850.0 pg/mg (0.0 pg/mg)*		
						topiramate	9.8 - 175.5 ng/mg (0.0 ng/mg)*		
						clobazam	186.5 - 4850 pg/mg (8.0 - 175.5 ng/mg) (0.0 pg/mg)*		
						clonazepam	5.6 - 8.4 ng/mg (0.0 ng/mg)*		
						phenobarbital	5.6 - 6.3 ng/mg (0.0 ng/mg)*		
						clozapine	31.7 ng/mg (0.0 ng/mg)*		
	38 postmortem samples	Cotton swab	Drying at room temperature for 24 h, solvent extraction	LC-TOF MS, LC-MS/MS	Drug abuse	Opiates, cannabinoids	2-100 ng/ 0.42- 8.2 mg cerumen	Blood, urine, hair, bile	Meier *et al.* (2017)
	24 females, 37 males (18-35 years)	Plastic curette	No sample pre-treatment	HS- GC/MS	Tobacco use/exposure	Nicotine	1.2 ± 0.5 ng/mg, passive smoker; 16.8 ± 32.9 ng/ mg, active smoker	Blood, urine, saliva, sweat, hair, nails	Shokry *et al.* (2017)
						Cotinine	5.3 ± 2.9 ng/mg, passive smoker; 25.4 ± 26.4 ng/ mg, active smoker		
						Anabasine	0.0 ng/mg, passive smoker; 326 ± 890 ng/ mg, active smoker		
						*o*-nicotine	3.7 ± 4.4 ng/mg passive smoker; 12.4 ± 17.4 ng/ mg, active smoker		
**Approach**	**Population** **tested**	**Sample** **collectionn**	**Sample** **pretreatment**	**Method of analysis**	**Disorder**	**Biomarkers detected**	**Detected ranges**	**Conventional bio-fluids**	**Authors**
**Chemical elements**	1 male, 1 female	clean Q tip	Desiccation, digestion in 10% nitric acid	Inductively coupled plasma atomic emission spectroscopy	Exposure to toxic elements	Lead	13.5 ± 0.71 μg/g (0.0 µg/g)*	Plasma, sweat, skin	Krishnan *et al.* (1992)
						Cadmium	1.14 ± 0.66 μg/g (0.0 µg/g)*		
	10 males, 10 females	ND	Incineration (for 6 hours) at 600^o^C	Flame Photometry	Fungal infection in the ear	Copper	0 ng/100gm (0.942- 3.314 ng/100gm)*	-	Yassin *et al.* (1966)
	4 male, 2 female (11-17 years), 1 male adult	Curette	Desiccation under vacuum, digestion in nitric/perchloric acid mixture (5:1 V/V)	Atomic absorption spectroscopy	Cystic fibrosis	Zinc	118 ± 103 μg/g (1857 ± 1341 μg/g)*	Blood, urine, saliva, sweat	Brand-Auraban *et al.* (1972)
	17 patients (7- 61 years)	ND	Incineration in a muffle furnace at 550 °C for 12 hours, dissolving in HCl	Clark-Collip method	Allergic Rhinitis	Calcium	6.87 ± 1.69 mEq/100 g (8.0 9± 1.86 mEq/100 g)*	Blood	Paiva *et al.* (1973)
				Fiske-Subbarow method		Phosphorus	26.9 ± 8.7 mg/100 g (18.63 ± 6.52 mg/100 g)*		
				Turbidimetric method		Sulfur	510.9 ± 152.7 mg/100 g (223.9 ± 35.1 mg/100 g)*		
				Diethyldithio-carbamate method		Copper	1.62 ± 0.49 mg/100 g (2.43 ± 1.29 mg/100 g)*		
	12 patients (46-72 years)	ND	Incineration in a muffle furnace at 550°C for 12 hours, dissolving in HCl	flame spectrophoto-metry	Cancer (prostate, mandible, tongue, tonsils, larynx)	Sodium	29.15 ± 8.11 mEq/100 g (39.5 ± 14.42 mEq/100 g)*	Blood, serum, urine, saliva, sweat	Paiva *et al.* (1973)
				Clark-CoUip method		Calcium	5.59 ± 1.76 mEq/100 g (8.09 ± 1.86 mEq/100 g)*		
				Yellow titan		Magnesium	1.84 ± 0.49 mEq/100 g (4.9 6± 2.56 mEq/100 g)*		
				Fiske-Subbarow method		Phosphorus	89.7 ± 15.1 mg/100 g (18.63 ± 6.52 mg/100 g)*		
	1 female (16 years), 2 males (22 and 28 years)	ND	ND	Histochemical examination	Wilson’s disease (hepatolenticular degeneration)	Copper	7.17 (2.43 mg/100 g)*	Serum, urine, faeces, cerebrospinal fluid, bile, saliva	Canelas *et al.* (1963)
	8 patients (24- 48 years)	ND	Incineration in a muffle furnace at 550 °C for 12 hours, dissolving in HCl	Clark-Collip method	Otosclerosis	Calcium	9.43 ± 3.03 mEq/100 g (8.09 ± 1.86 mEq/100 g)*	-	Paiva *et al.* (1973)
				flame spectrophotometry		Potassium	32.24 ± 4.17 mEq/100 g (37.67 ± 17.99 mEq/100 g)*		
1D-PAGE – one-dimensional polyacrylamide gel electrophoresis. LC-MS/MS – liquid chromatography/tandem mass spectrometry. DNA – deoxyribonucleic acid. PCR – polymerase chain reaction. RNA - ribonucleic acid. GC – gas chromatography. HS/GC-MS - headspace gas chromatography/mass spectrometry. DDT – dichlorodiphenyltrichloroethane. DDE – dichlorodiphenyldichlororthylene. HCB – hexachlorobenzene. HCH – hexachlorocyclohexane. UPLC-MS/MS - ultra performance liquid chromatography - tandem mass spectrometry. *Reference ranges for the biomarkers studied. *ND* - Not defined.									

#### Lipidomics

A great deal of effort was dedicated to the study of the lipid composition of cerumen, both casual and fresh as well as the variations detected with different earwax type, age, sex, season, menstruation *etc.* (*2*, *4*, *24*-*30*). The last approach was presented by Stransky *et al.* who performed a complete profiling of the cerumen lipid components in a sample (1.323 g) collected twice a week, throughout one year from both ears of a healthy 65 years old male (*17*). Then gradient column chromatography was used to separate cerumen into the aliphatic hydrocarbons, squalene, wax esters, cholesterol esters, triacylglycerols, free fatty acids, fatty alcohols, monoacylglycerols, cholesterol, sterols, and hydroxy acids which were then analysed separately, and identified by gas chromatography (GC) and gas chromatography/mass spectrometry (GC/MS) techniques.

Regarding the application of lipid analysis in cerumen, the majority of the studies of the lipid composition of cerumen were directed to the improvement of the developed ceruminolytic agents (*25*, *31*-*33*). This is due to the fact that the epidermal cells constituting approximately half of the mass of wax impactions are enveloped in a layer of bound lipids (w-hydroxyacids, free fatty acids, and ceramides) which contribute to the cellular cohesiveness which justifies why studying the lipid composition in specific could be useful in the choice and development of new ceruminolytics (*34*, *35*).

On the other hand, very little work was concerned with the use of cerumen lipids as diagnostic biomarkers in laboratory diagnostics. However, in this review, we tried to highlight some reports potentially relating cerumen lipids with some pathological conditions, either local (inside the ear) or systemic.

In 1954, Akobjanoff *et al.* identified some of the fatty acids in earwax (capric, lauric, oleic, myristic, linoleic, palmitic, stearic acids) as an approach to determine the normal constituents of cerumen (*36*). This may allow the detection of pathological conditions of the ear through changes from the normal. For instance, external otitis caused by a malfunction of the epidermal glands of the skin of the ear canal would be expected to show changes in the cerumen composition, which if recognized could be used for prophylaxis and treatment (*37*).

Later, Inabi *et al.* investigated the lipid composition in earwax of patients with hircismus using thin layer chromatography (TLC) (*38*). Samples were collected from 20 patients with hircismus (wet earwax type) and 20 adult volunteers without hircismus, extracted with n-hexane, and resolved by TLC using different solvents (hexane, benzene, ether, acetic acid). Spots were visualized by spraying with 50% sulfuric acid and charring at 220 °C and the unidentified fat was further analysed by GC and high performance liquid chromatography (HPLC). Results show that wet earwax is due to the difference in quantity and quality of earwax lipids and hircismus is associated with higher incidence of wax lipids. For instance, in wet earwax, steryl-esters and wax esters were not found as compared to dry earwax type while two unidentified lipids were found only in wet earwax type.

In 1966, a preliminary report undergone by a Japanese research group suggested a correlation between the lipid composition of earwax represented in the earwax type with the incidence of a coronary heart disease (arteriosclerosis). Based on the investigations performed on 96 Caucasian and Japanese arteriosclerotic in- and out- patients (with no reported age range), results showed that the incidence of wet cerumen among the patients with arteriosclerosis, not accompanied by hypertension, was strikingly high (30.2%), whereas it was 13.8% among arteriosclerotic patients with hypertension (*39*).

Later, in 1976, further investigations showed that Caucasian and Japanese populations’ dry cerumen contains 18% lipid and 43% protein, while wet cerumen has about 50% lipid and 20% protein. Since the cholesterol fraction of the lipid material is similar, the absolute amount of cholesterol excreted by persons with wet cerumen is inferred to be greater. This supports the assumption that the cerumen cholesterol concentration can give an indication about cholesterol concentration in blood (*40*). However, unfortunately, no further reports were found correlating cholesterol in cerumen with blood cholesterol. Moreover, modern methods to characterize lipids and lipoproteins do not seem to have been applied to cerumen and since it is conventional, in studies of disease association, to treat the first claim with due suspicion, therefore the relevance of cerumen types to lipid metabolism and arteriosclerosis remained an unresolved issue that can be neither asserted nor rejected (*40*).

Wet cerumen was also related to the incidence of *Tinea vescicolor* infection of the outer ear (*41*). Owing to its lipid composition, this type of earwax increases the susceptibility to lipophilic fungus “Malassezia furfur” responsible for *Tinea versicolor*. Two hundred and twenty three Japanese cases of *Tinea versicolor* were examined in Kumamoto, wet earwax was found in 90 cases (40.9%). These results indicate a significantly higher incidence of *Tinea versicolor* among individuals with wet earwax (*41*).

In another approach, 67 patients with Parkinson disease were subjected to the examination of their ear canals. By examination, the ear canals of one or both ears of 40 out of the 67 patients were found to be totally blocked with grossly excessive quantities of greasy hard wax (*42*). This is considered typical of the disease that causes increase in the activity of the sebaceous gland and thus the wax secretion. Psoriasis can also cause an increase in waxy material in the ear (*43*).

#### Proteomics

The composition of the protein in fresh and casual cerumen samples was first investigated photometrically by Chiang *et al.* (*25*). Later, an alpha-2-globulin was detected in cerumen by double diffusion and immune-electrophoresis but the first description of the isolation and quantification of total proteins of earwax was introduced by Schwaab *et al.* (*44*, *45*). Samples at an average weight of 77.75 mg were collected from ears of 16 healthy adults (wet earwax type) with a sterile hook under otoscopic control. Then, they were weighed, pulverized using a mortar and a pestle in liquid nitrogen. Proteins were then isolated by the Qproteome™ Mammalian Protein Prep Kit (Qiagen, Hilden, Germany) in two different kinds of ways (cell and lysate fraction). Afterwards, total protein concentration was quantified using the BCA protein assay kit (Thermo Fisher Scientific, Rockford, USA) method. This assay allows the colorimetric detection and quantitation of total protein using a unique reagent containing bicinchoninic acid.

The antimicrobial nature of cerumen has been investigated in relation to the levels of lysozyme and immunoglobulins present. Cerumen samples were collected by curette from 588 Caucasians, Black people, and Chinese; suspended in a buffer solution and mixed by a vortex mixer. Then, the lysozyme assay was performed as adapted from Osserman *et al.* while the antibodies were tested by immunodiffusion techniques set up with the cerumen suspension and immunoglobulin (Ig) A/IgE antibody (*46*, *47*). Lysozyme and immunoglobulins were present in almost all samples of the dry type but its frequency of occurrence varies significantly among the wet type depending on the race (*48*).

Lower lysozyme content and less acid pH in cerumen were also related to the occurrence of malignant otitis externa (MOE), an aggressive infection involving the external auditory canal and temporal bone, characterized by high mortality rate, aggressive disease progression and poor response to treatment (*49*).

Further study of the antimicrobial role of cerumen was carried out by quantitative estimation of 10 well known human antimicrobial peptides in earwax using enzyme linked immunosorbent assay (ELISA) (*50*).

Cerumen proteomic analysis was utilized for the first time as a non-invasive tool for biomarker analysis and disease diagnosis by Feig *et al.* employing liquid chromatographic-mass spectrometry (LC-MS) (*15*). A number of 11,562 distinct peptides representing 2013 proteins were identified in human cerumen. Five hundred and ninety-nine proteins (31%) were found unique to cerumen. Of these, 283 were successfully identified and by comparing the proportions of proteins in cerumen to multiple bio-fluids (saliva, urine and plasma), cerumen was found equally efficient as a novel bio-fluid in clinical diagnostics. In addition, the method allowed the detection of high amounts of zinc-alpha-2-glycoprotein, cathepsin D, apolipoprotein D, serpins, calpain, mucins and lysozyme C confirming the antimicrobial role of earwax. Thus, proteomic characterization of cerumen might provide explanations for local pathologies of the ear such as otomycosis, benign or malignant pathologies of the outer ear and susceptibility to recurrent infections such as chronic otitis externa, and can be applied for disease stage stratification as well.

Apart from that, proteomic characterization of cerumen could play a role in the diagnosis of systemic diseases where zinc-alpha-2-glycoprotein was already described to serve as either a potential biomarker for normo-albuminuric diabetic nephropathy, apocrine activity in breast cancer or a catabolic marker in cancer and noncancerous states (*51*-*54*). Apolipoprotein D is a lipoprotein related to increased total hydrophobicity and decreased susceptibility to infection by transport and binding to hydrophobic molecules, *e.g.* cholesterol esters, in the outer ear canal (*3*, *55*). An interaction of zinc-alpha-2-glycoprotein and apolipoprotein D also affects prolactin-inducible protein (a molecule regulating water transport in apocrine glands) (*56*). Prolactin-inducible protein is used as a potential marker for grading of apocrine carcinoma of the breast and interacts with IgG and CD4-T cell receptor (*57*).

Another glycoprotein was detected in cerumen, which is similar to salivary glycoprotein (EP-GP), a glycoprotein isolated from human saliva with homologues in several other body fluids (*58*). It was measured by quantitative ELISA in cerumen among other fluids and showed a wide variability while the EP-GP epitope bearing proteins were further characterized by electrophoresis and immunoblotting. The biological role of EP-GP is not exactly known but there were reports about its ability to bind different bacterial species both *in vivo* and *in vitro* (*59*).

#### Genomics

Cerumen was applied for the detection of different diseases using modern DNA testing techniques, such as for detection of chronic infection with hepatitis B (HBV) and ability to transmit hepatitis C viruses (HCV) (*60*-*62*). Detailed information on these applications is provided in Table 1.

An association between axillary odour and the wet-type earwax was first established only based on the phenotype more than 70 years ago (*7*). Later, this finding was confirmed using a SNP(rs17822931) of the *ABCC11* gene, the determinant gene of the earwax types, and furthermore was successfully used as a diagnostic marker for axillary osmidrosis (AO), a clinical condition of individuals with a deep anxiety regarding axillary odour and had undergone the removal of bilateral axillary apocrine glands (*7*). Further genetic association was found between wet earwax type, breast cancer (*63*), and AO (*64*). For the purpose of fast genetic diagnosis of AO and potential risk of breast cancer, specific primers were developed that allow to clinically genotype the *ABCC11* gene within 30 minutes (*64*). Further evidence was found on genetic association between wet earwax type, breast cancer, AO (*63*, *64*). Recently, a clinical method was developed to rapidly detect the genetic polymorphism (SNP 538G_A) in the *ABCC11* gene by a SmartAmp method in ≈ 30 min which not only enables fast diagnosis of AO but also potential risk of breast cancer genetically related to both wet earwax type and AO (*64*). Moreover, the SNP 538G_A responsible for the wet earwax type was further related to colostrum production from the mammary glands where the frequency of colostrum occurrence and its measurable volume are much higher among wet-type than the dry-type women. This could be important to provide anticipatory guidance for mothers about breast-feeding and the length of time that should be spent in feeding based simply on their earwax-type (*65*).

#### Metabolomics

##### Volatile organic compounds

Volatile organic compounds are a diverse group of stable carbon-based chemicals that are classified on the basis of their retention time and boiling point (ranging from 50°C to 260°C) (*66*).

Earlier, very little effort has been dedicated for analysis of VOC in cerumen, either alone (*21*) or along with other components as ash and electrolytes (sodium (Na), potassium (K), calcium (Ca), magnesium (Mg), and phosphorus (P)) to monitor the change in its content in association with some disorders as allergic rhinitis, otoschlerosis and cancer (*21*, *67*).

Recent studies have concentrated on the analysis of the volatile organic composition of earwax, as it is very lipophilic, and may act as an ideal substrate for retaining organic compounds indicative of physiological, dietary, environmental events and/or exposures and ethnic origins (*11*). The earwax VOC profile was indicative for some metabolic diseases as maple syrup urine disease (MSUD) and alkaptonuria, which were identified in earwax before being diagnosed using traditional techniques as blood and urine analysis (*68*-*71*).

Maple syrup urine disease was diagnosed by the characteristic burnt sugar odour that can be easily detected in patients’ earwax and in neonates < 5 days old. The smell is attributed to sotolone resulting from accumulation of branched chain AAs and 2-oxocarboxylic acids. On the other hand, alkaptonuria could be easily diagnosed at any age by the black earwax and the detection of homogentisic acid in samples by paper chromatography (*71*).

Most recently, cerumen was able to detect diabetes mellitus (DM) and to discriminate between its types 1 and 2 by monitoring of the changes in the volatile composition of samples collected from DM patients (types I and II) (*16*). Samples were analysed by headspace gas chromatography/mass spectrometry (HS/GC-MS) without previous extraction. Significant changes were obtained in the alcohols and ketones profiles, principally (ethanol, acetone, methoxyacetone, hydroxyurea, isobutyraldehyde, and acetic acid.

##### Amino acids

So far, AA analysis in earwax did not seem to have a role in medical diagnostics and the major part of it was dedicated to study the difference in AA composition between dry and wet earwax types as well as for development of new better ceruminolytic agents (*72*). For the later purpose, a study of AA composition of earwax as well as the carbohydrate content was done (*31*). The method employs an AA analyzer and LC with amperometric detection for AA and carbohydrate content, respectively. Glycine, glutamic acid, and serine were found to be the major AA components of earwax while galactosamine, galactose, glucose, glucosamine, mannose, and fructose were found in the carbohydrate part.

##### Carbohydrates

For medical diagnosis, studying the carbohydrate content in cerumen could be important as certain amounts of sugars in association with a nitrogen source and some AAs may prove advantageous to the growth of pathogens, and certain pattern of sugars in cerumen may be indicative of tumours and metabolic diseases (*31*). Only one abstract (in Russian) has been provided in this regard, about correlation of glucose concentration in cerumen with DM, both latent and manifest (*73*).

In addition, the carbohydrate content in cerumen was investigated for development of new ceruminolytic agents, by anion exchange column chromatography (CC) with pulsed amperometric detection and a gold working electrode. The carbohydrate analysis was performed on earplugs obtained from 10 patients that needed ear pug removal. The results reveal in the order of their abundance: galactosamine, galactose, glucose, glucosamine, mannose, and fructose with ratios of 2.4, 1.0, 0.9, 0.7 and 0.3, respectively (*31*).

#### Xenobiotics

Some studies suggest that earwax is like nails, hair and teeth may indicate chronic exposure since the last three media facilitate cumulative deposition of xenobiotics (*74*). Cerumen has been used for detection of long-term exposure to bio-accumulatory xenobiotics like lindane, chlordane, dichlorodiphenyltrichloroethane (DDT), dichlorodiphenyldichlororthylene (DDE), dieldrin, hexachlorobenzene (HCB), and hexachlorocyclohexane (HCH) which cause cancer mortality, using GC and GC-MS, as well as for detection of environmental exposure to metals (*74*-*76*). Despite that, the cerumen sampling is much simpler and acceptable than the surgical sampling of other adipose tissues, the results can only reflect cumulative exposure over a period of months or years rather than recent exposure and information relating to the chronic health effects of concern is lacking (*77*).

On the other hand, earwax was most recently used as a medium for monitoring drugs specially to indicate administration of drugs of abuse or drug facilitated crimes antiepileptics, anxiolytics, antipsychotics, *etc.* Cerumen could be even considered a more favourable surrogate to traditionally used biological fluids because of its non-invasiveness, ease of sample collection, minimum sample pretreatment, and relatively less external contamination in addition to being able to detect the analytes recently administered as well as drugs administered some months ago (*78*).

Further studies were extended to using post-mortem cerumen samples for detection of drugs of abuse, which may be correlated with the cause of death as opiates, amphetamine and derivatives, cocaine, methadone and/or derivatives (*79*). Samples were collected using cotton swabs, dried at room temperature for 24 hours before extraction and analysis by (liquid chromatography/time of flight mass spectrometry (LC-TOF MS) and LC-MS/MS (*79*).

It was also applied for detection of tobacco use/exposure by the monitoring nicotine and its related compounds (cotinine, anabasine and *o*-nicotine). Moreover, it was able to distinguish non- or passive exposure to tobacco smoke from active exposure. Samples of 20 mg were collected from 61 young adults (18-35 years) using a plastic curette and analysed directly by HS/GC-MS without previous extraction. Cotinine and anabasine were found to be the biomarkers capable of discriminating completely between the study groups due to the significant difference in their detected concentrations (*80*).

#### Chemical elements

In 1992, Krishnan *et al.* presented the first report of the use of earwax as a biological monitoring medium for metals (*81*). Thirty-eight elements were analysed in cerumen samples obtained from one Eurasian male in his middle forties and one female from the Indian subcontinent in her thirties, by inductively coupled plasma atomic emission spectroscopy (AES). Results demonstrated the non-detection of silver (Ag), boron (B), beryllium (Be), cobalt (Co), mercury (Hg), manganese (Mn), nickel (Ni), selenium (Se), and vanadium (V) which suggests potential usefulness of earwax as a biological monitoring medium for these toxic elements in people exposed to high concentrations in the environment or in the workplace, since no baseline correction is required unlike for the other elements. On the other hand, Pb and Cd were detectable in both samples which signifies possible use of earwax to assess their external exposure.

Later, several studies were carried out that related the metal content to the health status and pathological conditions as cystic fibrosis, allergic rhinitis, otoschlerosis, cancer, and Wilson’s disease (*82*-*84*). For instance, cystic fibrosis patients show lower concentrations of all the detected electrolytes (Na, K, Ca, Mg, Cu, Zn), principally the later (*82*).

It was also indicative of ear infections where the fungal growth in the ear was investigated in relation to the content of iron and copper amounts detected in earwax samples obtained from 10 male and 10 female Egyptians (*85*). Samples were collected in Pyrex glass sterile tubes and micro-chemical analysis was carried out by flame photometry. High copper concentrations were found in samples of some subjects indicating the absence of fungal infections while iron, which is also toxic for fungal growth, was undetectable in all the experimental samples.

The metal content of cerumen was also used as a method to study the pathogen biodiversity of human cerumen by using an optical probe for metal content characterization (*86*).

## Future perspectives of cerumen analysis

A great effort needs to be done to incorporate earwax diagnostics into daily use where collection methods and biomarkers need to be standardized and validated. Prospective specimen collection and retrospective blinded evaluation are typically used for this purpose to minimize bias and reinforce significance. To fulfil these protocols prior to diagnosis, large patient populations, procurement and categorization of their samples, and clinical information are required. Cerumen is assessed quantitatively to determine the specificity, sensitivity, and reproducibility of the biomarker(s) in question. Further evaluations are also required to explore the capability of detecting and accurately measuring the markers in relatively low concentrations. Then before cerumen is used in a clinical assay, it is subjected to five stages including: 1) preclinical testing where biomarkers are discovered in patient samples and confirmed either *in vitro* or *in vivo*; 2) feasibility analysis in which biomarkers are tested using small patient subpopulations to demonstrate their ability to detect disease; 3) validation process, which involves accurate testing for biomarkers; 4) statistical analysis to verify if statistically significant differences were obtained in a large patient population; 5) investigating the biochemical functions of the biomarker as understanding of the molecular mechanisms of biomarkers enables them to be more informative of the disease, progression and potential treatments. In addition, new assays and devices need to be developed at a commercially feasible rate. This could make earwax-based diagnostic tests more accepted by health care professionals, consequently facilitating the generation of further studies to demonstrate and establish the accuracy, sensitivity, and specificity of earwax diagnostics in a much wider variety of diseases. This involves studying for instance, the impact of interindividual variations (*e.g.* age, gender, heath status, racial differences, *etc.*), the time course of the investigated biomarkers in earwax, and correlation of its levels with corresponding levels in blood and/or urine. Accomplishing this along with the establishment of defined guidelines for the procedures might make earwax diagnostics a reality in the future especially that there has been increasing applications of earwax analysis in forensics as evident in the recent literature. In this review, we highlighted some advantages and limitations of earwax as a diagnostic bio-fluid as shown in Table 2.

**Table 2:** Advantages and limitations of earwax testing for laboratory diagnostics

**Advantages**	**Limitations**
Noninvasive, easy to collect, low cost	Despite its accessibility, it has not been widely and sufficiently studied as a bio fluid
No/minimum external contamination	Earwax composition shows a high inter-individual variability depending on many factors (sex, age, season, menstruation, *etc.*)
No risk of disease transmission as in blood sampling	Timely reproduction, since time (few days) is required for the build-up of the sample thus it is not suitable for continuous monitoring
No need for trained medical staff	
Samples are easy to ship and store	
No/minimum embarrassment or discomfort associated with blood and urine tests	Lack of standardized methods for earwax collection
Requires less sample pretreatment or manipulation for diagnostic tests	
Can detect both recent and long-term exposure unlike blood, urine, saliva, *etc.*	Till now, cerumen analysis involves complicated instrumentation as GC–MS, LC-MS that requires trained personnel
Economical sampling, shipping and storage compared to blood	
Sampling can be done at home	
GC-MS – gas chromatography - mass spectrometry. LC-MS - liquid chromatography-mass spectrometry.	

## Conclusion

This review provides a summary of the biology, functions of cerumen, past and current investigations performed to establish biomarkers that could be potentially applied in disease detection combined with noninvasive sample collection. It also focuses on the potential role of earwax as medium for biological monitoring and a new frontier for medical diagnosis and forensic applications highlighting its advantages in comparison to traditional diagnostic tests. It also encourages further research on earwax as a promising alternative biological fluid. Some limitations were found in our review, where relatively less data was available on applications of cerumen in medical diagnosis and some of the references could be somehow outdated. That is probably due to the fact that earwax until recently have been looked upon as a neglected body secretion and thus many years were needed to reach a considerable amount of data supporting the diagnostic potential of earwax.
